# High-resolution laser resonance ionization spectroscopy of $^{143-147}$Pm

**DOI:** 10.1140/epja/s10050-020-00061-8

**Published:** 2020-02-24

**Authors:** Dominik Studer, Jiri Ulrich, Saverio Braccini, Tommaso Stefano Carzaniga, Rugard Dressler, Klaus Eberhardt, Reinhard Heinke, Ulli Köster, Sebastian Raeder, Klaus Wendt

**Affiliations:** 1 Institut für Physik, Johannes Gutenberg-Universität Mainz, 55099 Mainz, Germany; 2 Paul-Scherrer Institut, 5232 Villigen, Switzerland; 3 Albert Einstein Center for Fundamental Physics, Laboratory for High Energy Physics, University of Bern, 3012 Bern, Switzerland; 4 Institut für Kernchemie, Johannes Gutenberg-Universität Mainz, 55099 Mainz, Germany; 5 Institut Laue-Langevin, 38042 Grenoble, France; 6 Helmholtz-Institut Mainz, 55099 Mainz, Germany; 7 GSI Helmholtzzentrum für Schwerionenforschung, 64291 Darmstadt, Germany

## Abstract

We present the results of high-resolution laser spectroscopy of the long-lived radioactive isotopes $^{143-147}$Pm. The hyperfine structures and isotope shifts in two different atomic ground-state transitions at 452 nm and 468 nm were probed by in-source laser spectroscopy at the RISIKO mass separator in Mainz, using the PI-LIST ion source. From the hyperfine coupling constants the nuclear magnetic dipole and electric quadrupole moments for $^{143-147}$Pm were derived, and the measured isotope shifts allowed the extraction of changes in nuclear mean square charge radii.

## Introduction

High-resolution laser spectroscopy of atomic transitions can be used as a high precision, model-independent probe for a number of fundamental properties of nuclear ground states or long-lived isomers. The analysis of hyperfine splittings allows the extraction of nuclear spin, magnetic dipole moment and electric quadrupole moment, while isotope shifts are linked to changes in mean square charge radii along a series of isotopes [1–3]. During the last decades, with the use of radioactive ion beam facilities based on the Isotope Separation On-Line (ISOL) technique in combination with sensitive laser spectroscopy methods, such studies continued to push further away from the valley of beta-stability, towards very exotic short-lived radioisotopes. In this regard the region of lanthanide elements is one of the most thoroughly studied along the entire chart of nuclei. Promethium (Pm, $Z=61$), however, marks a gap in the map of investigated nuclei [1], which can be attributed to its exclusively radioactive nature and its complex atomic spectrum, rendering preparatory experiments difficult.

Precision spectroscopy in the Pm isotopic chain is of high relevance to gain information on nuclear moments and for the study of nuclear shape transition phenomena. Leander et al.  expect a transition from spherical nuclei to a regime of strong deformation towards neutron deficient isotopes in the light lanthanide region, which is predicted to be best accessible (at $N < 75$) and particularly sharp in the case of Pm [4]. On the neutron-rich side, the shape transition to deformed nuclei for $N>88$ can be studied. The influence of $^{146}$Gd, which shows certain features typical for a doubly magic nucleus [5], has been related to the abrupt change in charge radii in this region. Budick et al.  observe a remarkable degree of deformation in $^{151}$Pm compared to $^{147}$Pm, measured via atomic beam magnetic resonance (ABMR) [6], similar to what has been observed for the corresponding isotones of Eu [7]. Although we cannot access these neutron numbers in our off-line experiment, a valuable basis for on-line studies can be established.^1^

Modern cyclotrons are capable of producing a number of long-lived Pm isotopes in relevant quantities and with suitable specific activity, rendering laser spectroscopic experiments feasible. In the historical context Pm spectroscopy is not entirely new, however, experiments were most often limited to the easiest accessible isotope, $^{147}$Pm. First hyperfine patterns were measured in the 1960s by Klinkenberg et al.  [8] and Reader et al.  [9]. In these experiments, milligram amounts of $^{147}$Pm were used in both, electrodeless discharge or hollow cathode light sources and studied using grating-based and Fabry–Pérot spectrographs. Although several hundred lines were measured, the assignment of the associated energy levels was not possible in most cases, and sometimes even the information was lacking whether a specific line belongs to the spectrum of neutral (Pm I) or singly ionized (Pm II) promethium. Nonetheless, a nuclear spin of $I=+7/2$ and nuclear moments of $\mu _I= 2.58(7)\,\mu _N$ and $Q_s=0.74(20) \, e\text {b}$ for $^{147}$Pm could be extracted, which are the most precise values until today (together with values obtained from complementary measurement methods). First direct excitation spectroscopy was performed in the 1990s by Alkhazov et al.  [10] and Otto et al.  [11] by means of collinear fast beam laser spectroscopy using dye lasers. In both experiments transitions in the spectrum of Pm II were studied. Alkhazov et al.  also had $^{145}$Pm at their disposal, which was produced in the reaction $^{144}\text {Sm}(n,\gamma )^{145}\text {Sm}(\text {EC})^{145}\text {Pm}$, and accordingly also extracted nuclear moments for this nuclide, with the precision limited by the reference nuclear moments in $^{147}$Pm [12].

Other than these laser spectroscopic studies on Pm II transitions, our work is dedicated to the study of Pm I. In the scope of our recent work on the atomic structure of neutral Pm, we identified several laser ionization schemes and determined the first ionization potential of Pm [13]. Utilizing these schemes, two atomic ground state transitions at $452\,\text {nm}$ and $468\,\text {nm}$ are investigated here. In contrast to many state-of-the-art spectroscopy experiments based on collinear laser spectroscopy of fast atom- or ion beams, we performed in-source spectroscopy directly in the atomic beam effusing from a hot atom source. This concept is implemented in the PI-LIST (perpendicularly illuminated laser ion source and trap), which presents a complementary technique to collinear laser spectroscopy and has undergone various performance tests on stable and radioactive species lately [14].

## Experimental setup

### Sample production and purification

The samples for our experiment originate from two different production routes. One sample, containing some $10^{14}$ atoms of $^{147}$Pm, was produced by neutron activation of enriched $^{146}$Nd at the high-flux reactor at ILL Grenoble and purified at PSI Villigen. For details on the production we refer to [15]. Part of this sample was already used for our studies of the atomic structure of Pm [13]. Other suitable isotopes for off-line experiments are $^{143, 144, 145, 146, 148\text {m}}$Pm, with half-lives of at least some 10 of days, which is required for chemical purification and shipping. To complement the isotopes accessible by neutron irradiation we opted for proton irradiation of a natural neodymium target, which was performed at the 18 MeV proton cyclotron at Bern University Hospital [16]. A target pellet with $1 \, \text {cm}$ diameter and thickness of $0.65 \, \text {mm}$ was pressed from a mixture of natural Nd$_2$O$_3$ and graphite powder with a total weight of $113 \, \text {mg}$. The addition of approx. 25 wt% graphite as binding agent was necessary to increase the mechanical stability of the pressed pellet, which was then encapsulated in an aluminum sample holder and irradiated with an integrated current of approximately $12 \, \upmu \text {Ah}$. After irradiation, the pellet was removed from the aluminum holder, the Nd$_2$O$_3$ was dissolved in 7M HNO$_3$ and the graphite was removed by filtration. The radiochemical separation of the produced Pm isotopes from the Nd bulk material was performed by ion exchange chromatography on the SYKAM cation exchange resin, following the procedure described in [15]. As a tracer of the Nd-fraction during the chemical separation, 1 MBq of $^{147}$Nd was produced by neutron activation of a natural Nd$_2$O$_3$ solution in the TRIGA research reactor at the Department of Nuclear Chemistry at Mainz University, and afterwards shipped to PSI Villigen, where the radiochemical separation took place. An ICP-MS analysis of the Pm fraction was performed after separation. A Nd:Pm ratio of approx. 100:1 indicates a decontamination factor of Pm from Nd of around $6\cdot 10^5$. A separate publication with detailed information on the production and separation is in preparation, in which also half-life measurements of $^{143,144}$Pm will be presented [17].

**Table 1:** Composition of the Pm sample produced by irradiation of a Nd$_2$O$_3$ target with $12 \, \upmu \text {Ah}$ of $18 \, \text {MeV}$ protons. Half-lives were taken from [18]. The activity and atom number *n* were determined by $\gamma$-spectroscopy. The mass ratio was measured via RIMS (see Fig. 1) 19 days after production

Nuclide	$T_{{1}/{2}}$	Activity (kBq)	$n/10^{12}$	Mass ratio (%)
$^{143}$Pm	$265(7) \, \text {d}$	60.7(7)	2.00(6)	17.2(15)
$^{144}$Pm	$363(14) \, \text {d}$	84.9(8)	3.8(2)	35.2(24)
$^{145}$Pm	$17.7(4) \, \text {y}$			17.6(15)
$^{146}$Pm	$5.53(5) \, \text {y}$	6.9(2)	1.74(6)	18.7(16)
$^{147}$Pm	$2.6234(2) \, \text {y}$			9.9(11)
$$^{148}$$Pm	$$5.368(7) \, \text {d}$$	116(2)	0.077(1)	$$1.1(3)$$$$*$$
$^{148\text {m}}$Pm	$41.29(11) \, \text {d}$	18.1(2)	0.093(1)	

The value of 1.1(3)% marked with an asterisk gives the combined mass ratio for $^{148}$Pm and $^{148\mathrm{m}}$Pm

Table 1 comprises all Pm nuclides which were produced in relevant quantities. Half-lives are taken from the Evaluated Nuclear Structure Data File (ENSDF, [18]). The activity was measured via $\gamma$-spectroscopy at PSI Villigen, and the atom number of each nuclide *n* was derived from the $\gamma$-activity. No $\gamma$-lines for $^{145}$Pm and $^{147}$Pm could be observed in the $\gamma$-spectra and thus no activity and atom numbers could be determined by means of $\gamma$-spectroscopy for these isotopes. This is expected as both isotopes have significantly longer half-lives, resulting in lower emission rates of their decay radiation in the sample. Furthermore, the $\gamma$-ray emission probabilities during the decay of both isotopes are low in general (especially for $^{147}$Pm they are well below $10^{-4}$). The possibly detectable line of $^{145}$Pm at $72.5 \, \mathrm {keV}$ is obscured in the measured spectrum with the much more prominent $K\alpha _2$ line of lead at $72.8 \, \mathrm {keV}$, originating from X-ray fluorescence of the detector shielding. For an additional analysis of the sample composition, mass spectra were recorded via resonance ionization mass spectrometry (RIMS). The RIMS measurements were performed 19 days after the $\gamma$-spectroscopy (details on this measurement are discussed in Sect. 2.3). While RIMS itself does not give information about absolute atom numbers, the isotope ratios can be compared with the ones from $\gamma$-spectroscopy. The ratios match within the uncertainties (with consideration of the decay time), so we can conclude that atom numbers of $^{145}$Pm and $^{147}$Pm are also in the order of $10^{12}$ atoms, similar to the other long-lived isotopes $^{143, 144, 146}$Pm.

### Laser setup

Our Pm laser ion source relies on two different laser ionization schemes, which we developed in our previous work [13]. Both schemes use three laser steps $\lambda _1, \lambda _2, \lambda _3$ to consecutively excite sample atoms to higher lying atomic states, with the final state having an excitation energy above the first ionization potential and thus undergoing auto-ionization. 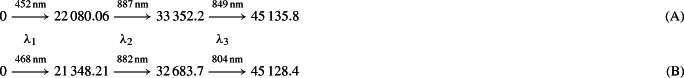 All level energies are given in units of $\, \text {cm}^{-1}$.

By measuring the number of produced ions as a function of the laser wavelength, spectroscopy can be performed. The ionization schemes will be abbreviated in the following by (A) and (B), respectively, where $\lambda _1$ is the spectroscopy transition in both schemes. Each step was driven by a $10 \, \text {kHz}$ repetition rate pulsed Ti:sapphire laser, with pulse lengths of 40–60 ns, an average output power of 3–4 W, and a spectral linewidth of 5–10 GHz. For a detailed description of these home-built “Z-cavity” lasers, which are in use at on-line radioactive ion beam facilities worldwide, see e.g. [19, 20]. Since $\lambda _1$ is in the blue wavelength regime, we generated the second harmonic intra-cavity, using a beta barium borate (BBO) crystal. For measuring hyperfine spectra we can alternatively produce $\lambda _1$ by an injection-locked Ti:sapphire laser with a Fourier-limited linewidth of $\approx 20 \, \text {MHz}$ for the spectroscopy transition [21, 22], seeded by an external cavity diode laser (master ECDL). In this case the second harmonic was generated outside the cavity by focusing the laser into a BBO crystal in simple single-pass transmission. For the master ECDL we used two different laser diodes: Eagleyard RWE-920 and RWE-980 for scheme (A) and (B), respectively. It was stabilized with an iScan unit (TEM Messtechnik GmbH). For a relative laser frequency measurement we simultaneously recorded the output of the master ECDL and a stabilized HeNe laser (SIOS SL-03) in a home-built scanning Fabry–Pérot-interferometer (S-FPI) with a free spectral range of $299.721 \, \text {MHz}$ and a finesse of $\mathcal {F}\approx 400$. The frequency offset to an arbitrary anchor point can be deduced from the distance of the transmission fringes of the master ECDL laser when using the transmission fringes of the HeNe as a ruler. For a complementary, absolute frequency measurement we used a wavelength meter (High Finesse WSU-30). An additional ECDL (Toptica DL pro 780), locked to a Rb saturation absorption spectroscopy unit (TEM CoSy 4.0) served as a calibration source for the wavelength meter. Note that in a comparative study of the wavelength meter and the S-FPI readout, performed at different laboratories, we observed periodic deviation patterns which necessitate a correction to recorded spectra [23]. The data presented here was corrected for this periodic behavior by subtracting a frequency deviation term, according to Eq. 1 in [23], from the wavelength meter data. A drift correction was not applied to the data, but rather a frequent calibration of the wavelength meter to the reference laser (see section IIIB in [23]). The reference also provides a more detailed description of the cw-laser setup.

### Ion source setup

**Fig. 1:**
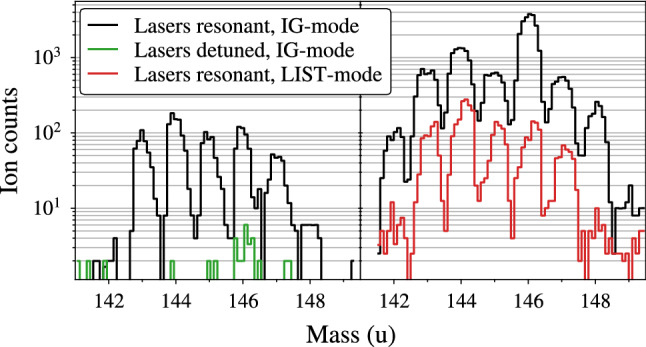
Mass spectra of the cyclotron-produced Pm samples. Left panel: Cold ion source. Right panel: Hot ion source at $\approx 1500~~^{\circ }\text {C}$. For details see text

The laser spectroscopy was performed at the RISIKO mass separator at JGU Mainz, using the resonance ionization spectroscopy technique. The used setup, i.e. its standard configuration, is described in [24]. The sample solution was dried on a titanium carrier foil, folded and put into a tubular tantalum atomizer, which can be resistively heated up to $2000~~^{\circ }\text {C}$. For the experiment we used a refined version of the well-proven Laser Ion Source and Trap (LIST) [25–27]. It features a dual repeller electrode on the side facing the atomizer oven, a rf quadrupole for radial confinement of ions, and an exit electrode to prevent field leakage of the strong extraction potential in the LIST volume. It has two modes of operation. When operated in ion-guide (IG) mode both repellers are set on a negative voltage, so that positive ions are extracted from the source. The ions are guided towards the exit electrode by the rf field. Upon passing the exit electrode, they are accelerated to $30 \,\text {keV}$ (from the source potential at + 30 kV towards the grounded extraction electrode). The IG mode resembles the standard laser ion source operation without a LIST unit, with an efficiency loss factor of $< 2$ [25]. In the second mode of operation (LIST mode) one repeller is set on a positive voltage, so that ionized species from the source region (e.g. via surface ionization) are suppressed. The negative repeller electrode deflects electrons emitted from the hot oven, preventing electron impact ionization within the LIST volume. The LIST mode thus offers a suppression of ions which are produced independently of the laser, in particular isobaric contaminants which cannot be mass-separated. Since laser-ionized species from the source region are lost, this gain in ion beam purity comes at the cost of ionization efficiency. Despite this trade-off this technique offers unique opportunities whenever the ion beam composition is dominated by isobars of the nuclides of interest, which cause strong background. In the present experiment this was the case for e.g. Nd isotopes from the cyclotron target. Figure. 1 shows mass scans in the Pm region for different ion source conditions. For these scans we used the broadband laser system tuned to scheme (A), so that full hyperfine splittings and isotope shifts were covered by the laser linewidth and all Pm isotopes were equally ionized. In the left panel, no heating current is applied to the atomizer and the LIST is operated in IG-mode. The release of sample atoms was caused by heating of the atomizer by the incident laser beams. In this situation the influence of the laser ionization scheme could be well tested, as the contribution of surface ionization was negligible. We observe the isotope ratio as expected from the $\gamma$-spectroscopy measurements given in Table 1, with a slight interference of about 3 % intensity at mass 146, occurring when the lasers were detuned from the Pm ionization scheme. In the right panel, at a temperature of approximately $1500~~^{\circ }\text {C}$, the measured isotope ratios in IG-mode are significantly different. The dominant components of the surface ionized pattern are expected to be atomic neodymium from the cyclotron target and a so-far unidentified species on mass 146. The latter was weakly influenced by the $452 \, \text {nm}$ laser radiation and shows a pattern of equidistant resonances with a $\approx 4.2 \, \text {GHz}$ spacing. We therefore presume a molecular species (possibly in a higher charge state) where a vibrational band was excited by the laser light, and which was subsequently ionized non-resonantly by a second photon.

**Fig. 2:**
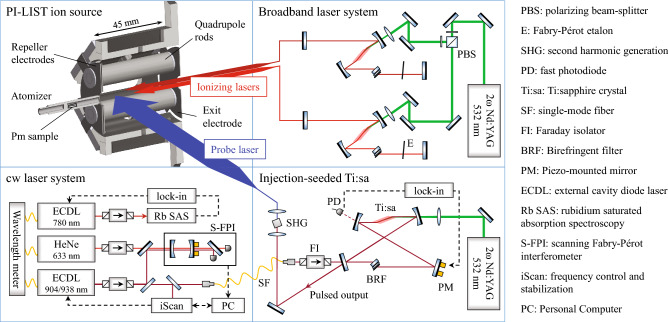
Sketch of the experimental setup. Top left: vertical cross section of the PI-LIST ion source unit with indicated incident laser beams. For the sake of clarity the mounting and heat shielding of the resistively heated atomizer is not shown. Top right: broadband pulsed Ti:sapphire lasers for ionizing transitions. Bottom left: cw laser system with seeding diode laser and frequency measurement references. Bottom right: pulsed, injection-seeded Ti:sapphire ring cavity for spectroscopy transition. A legend for the used abbreviations is given on the right

When switching to LIST-mode, these contaminants were suppressed and the ion beam composition was similar to the one of the cold ion source. Isotope ratios were measured with both, a cold source in IG-mode and with the hot source in LIST-mode in good agreement, mean values are given in Table 1. In the following, for the high-resolution spectroscopy application, the ion source was exclusively operated in LIST-mode, introducing the injection seeded probe laser for the spectroscopy transition in perpendicular geometry [14]. The probe laser beam was horizontally widened with a profile of approximately $40 \, \text {mm} \times 2 \, \text {mm}$ for a large overlap area with the ionizing lasers. A sketch of the experimental arrangement is given in Fig. 2.

**Fig. 3:**
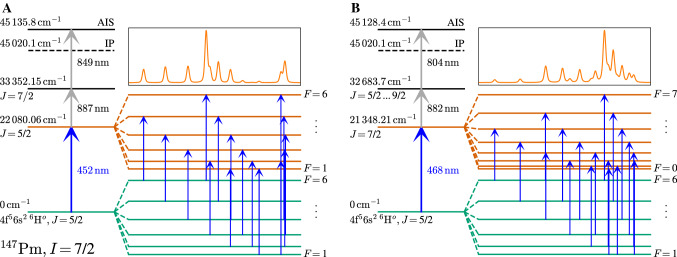
Excitation schemes (**A**) (left) and (**B**) (right) illustrating the hyperfine splitting of the atomic ground state and respective first excited state. The blue arrows indicate allowed hyperfine transitions, with the positions chosen in relation to the example spectrum presented above. For the spectrum we chose the fit function to our $^{147}$Pm data. IP: Ionization potential; AIS: Auto-ionizing state

## Hyperfine spectroscopy

**Fig. 4:**
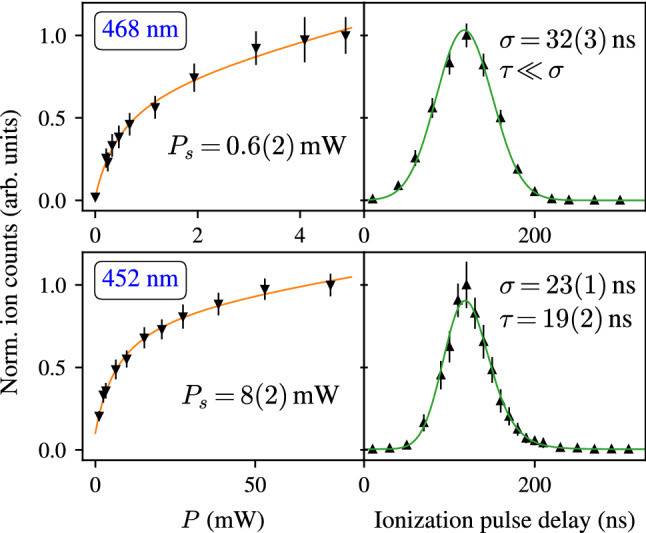
Laser power (left) and ionization pulse delay (right) influence on the ion signal for the transitions at $468 \, \text {nm}$ and $452 \, \text {nm}$. Saturation curves (orange) are fitted according to [28]. The lifetime is fitted with a convolution of a Gaussian distribution with an exponential decay law (green). The zero-point of the ionization pulse delay is arbitrary

The hyperfine splittings of both spectroscopy transitions, at $452 \, \text {nm}$ and $468 \, \text {nm}$, are schematically illustrated in Fig. 3. The positions and spacings of the arrows indicating individual hyperfine transitions are chosen in such a way that they depict the position of the respective line in the spectrum, as plotted exemplarily for the case of $^{147}$Pm. In order to achieve a sufficiently narrow experimental linewidth to resolve the hyperfine patterns, several parameters which are specific to the ionization scheme were characterized. Firstly, the power of the spectroscopy laser has to be properly chosen to prevent saturation broadening of the spectral lines. The laser power influence on the two spectroscopy transitions of interest was measured, and is shown in the left panels of Fig. 4. In scheme (A) we chose the $F=6 \rightarrow F'=5$ transition and in scheme (B) the $F=5 \rightarrow F'=6$ transition between the ground state and the respective first excited state. A fit according to the procedure described in [28] yields saturation powers (defined as the power at which half of the maximum ion signal is reached) of $P_s^{452} = 8(2)\,\text {mW}$ and $P_s^{468} = 0.6(2)\,\text {mW}$ for the transitions at $452 \, \text {nm}$ and $468 \, \text {nm}$, respectively. As not all components of the hyperfine spectrum were investigated and the values are specific to the individual hyperfine transitions, these values are used as guide figures to estimate the power threshold upon which saturation broadening occurs. In the earlier broadband spectroscopy experiment [13] we measured a comparable saturation power of $P_{s, \text {ref}}^{452} = 7(4)\,\text {mW}$ in the first step of scheme (A). In scheme (B), however, the earlier measured value of $P_{s, \text {ref}}^{468} = 10(5)\,\text {mW}$ deviates by an order of magnitude from the value in this work, which is ascribed to different operation conditions, i.e. a change of the laser power density by a variation in the laser beam profile, as well as the relatively high intensity of the $F=5 \rightarrow F'=6$ transition in the pattern of the $468 \, \text {nm}$ transition. For hyperfine spectroscopy in the $452 \, \text {nm}$ and $468 \, \text {nm}$ transitions, the first excitation laser were correspondingly operated at $5 \, \text {mW}$ and $0.5 \, \text {mW}$, respectively.

Another spectral line broadening effect is caused by the high-power ionization lasers, which couple the excited state to the ionization continuum. When the probe and the ionization lasers are synchronized, the lifetime of the excited state is effectively shortened, causing a line broadening [29]. In order to avoid this effect, the ionization laser has to be decoupled from the excitation step by temporal delay. However, depending on the excited state lifetime, the population decay causes a certain loss in efficiency. The loss factor was measured for the transitions of interest by simultaneous and stepwise shifting the second- and third laser ($\lambda _2$, $\lambda _3$) pulse delays relative to the spectroscopy excitation. Since the probe laser is pumped by a separate pump laser, the second- and third laser pulses can be delayed simultaneously by adjusting the pump laser triggers relative to each other. The probe laser was tuned to the same hyperfine transitions as used in the measurement of saturation powers, with a power of $70 \, \text {mW}$ in the $452 \, \text {nm}$ transition and $5 \, \text {mW}$ in the $468 \, \text {nm}$ transition. The response of the ion signal is shown in the right panels of Fig. 4. It can be fitted with a convolution of the approximately Gaussian laser pulse shape with an exponential decay contribution for the lifetime of the excited state [30]. In the case of the $452 \, \text {nm}$ transition, a lifetime of $\tau _{452} = 19(2)\,\text {ns}$ is extracted for the upper state. For the $468 \, \text {nm}$ transition, however, the signal shape is completely dominated by the Gaussian contribution. The larger Gaussian standard deviation compared to the measurement in the $452 \, \text {nm}$ transition is caused by the laser operation near the edge of the Ti:sapphire gain profile, leading to an extended laser pulse length. As a consequence the lifetime of the excited state of the $468 \, \text {nm}$ transition can only be constrained to be significantly shorter than the laser pulse length. This finding is consistent with the much lower saturation power compared to the $452 \, \text {nm}$ transition. For the spectroscopy experiment a delay between 30 and $50 \, \text {ns}$ was chosen for both transitions, as a reasonable compromise between linewidth and efficiency. In the $452 \, \text {nm}$ transition, we measured an efficiency loss factor of $\approx 6$ for a delay of $30 \, \text {ns}$ and a laser power of $5 \, \text {mW}$, while the linewidth improved to a value of $\approx 150 \, \text {MHz}$ full width at half maximum (FWHM), down from $\approx 250 \, \text {MHz}$ FWHM as determined without delay and with $\approx 70 \, \text {mW}$ laser power.

**Fig. 5:**
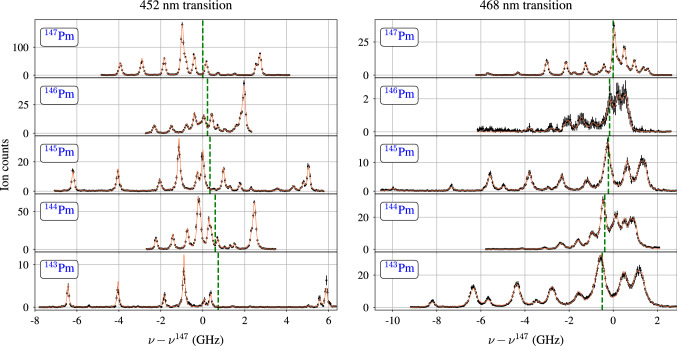
Measured hyperfine spectra in the ground state transitions of scheme (**A**) at 452 nm (left) and of scheme (**B**) at 468 nm (right). The centroid frequency of the $^{147}$Pm hyperfine structure is taken as reference for the frequency offset $\nu - \nu ^{147}$, which is indicated by the green dashed line. Fit parameters are given in Table 2

Scans of the hyperfine spectra were performed by tuning the ECDL master laser in steps of $10 \, \text {MHz}$ while recording the ion signal. Depending on the counting statistics for the measured isotope, data was taken for 3–5 s per step, so that one spectrum took approximately 1–2 h to record. The wavelength meter was calibrated to the Rb-locked ECDL every 10 steps in order to keep drifts of the wavelength readout at minimum. This is particularly important since one isotope at a time is measured, and the accuracy of extracted isotope shifts relies on the reproducibility of the data. The recorded spectra are shown in Fig. 5. For all isotopes, with the exception of $^{143}$Pm, at least two datasets could be recorded per isotope and transition. In the $452 \, \text {nm}$ transition the spectral resolution varies between 100 and $170 \, \text {MHz}$ FWHM, whereas the $468 \, \text {nm}$ spectra are somewhat inferior with regard to counting statistics and linewidths lying between 150 and $250 \, \text {MHz}$ FWHM. The spectra were fitted with a sum of Voigt profiles, using the SATLAS python package [31], which is tailored to the evaluation of hyperfine spectra. The nuclear spins of each isotope could be fixed to literature values ($^{143}$Pm: [32], $^{144}$Pm: [33], $^{145}$Pm: [10], $^{146}$Pm: [34], $^{147}$Pm: [10, 35], with the values comprised in Table 3). In order to estimate the statistical uncertainties, all datasets were fitted with both, the wavelength meter and the S-FPI laser frequency data, and for three different binning sizes. The resulting fit parameters were averaged and the standard deviation taken as uncertainty. Afterwards, for different datasets of one isotope, a weighted average was determined. For the isotope shifts we added a systematic error of $4 \, \text {MHz}$, based on the stability of the $^{87}$Rb saturated absorption spectroscopy, which serves as reference for the wavelength meter. In both transitions, the data for the $^{147}$Pm isotope has the highest quality in terms of linewidth and counting statistics, since the scan was performed with a dedicated sample with larger atom numbers ($\approx 3 \times 10^{12}$ atoms), whereas the cyclotron-produced samples were comparatively small (few $10^{11}$ atoms) and suffered a larger Nd contamination. Additionally, $^{147}$Pm has the highest nuclear quadruple moment $Q_s$ among the studied isotopes. For these reasons it was used as a reference for the ratio of the electric quadrupole hyperfine coupling constant of lower and upper level, i.e. $\mathcal {B}^l/\mathcal {B}^u$, which is expected to be constant over the series of isotopes [1]. Correspondingly, in the fits of both transitions, this ratio was fixed to the result of $^{147}$Pm in the SATLAS fit of the other isotopes. Also, for reasons of superior data quality in the $452 \, \text {nm}$ transition, $\mathcal {B}^l_{468}$ was fixed to $\mathcal {B}^l_{452}$, since both transitions couple to the atomic ground state. The magnetic dipole hyperfine coupling constants $\mathcal {A}_l$ and $\mathcal {A}_u$, on the other hand, remained a free parameter in all fits.

**Table 2:** Extracted parameters from the hyperfine spectra of the $452 \, \text {nm}$ and the $468 \, \text {nm}$ transitions. The superscripts *l* and *u* denote the associated lower and upper level of the respective transition. Isotope shifts $\delta \nu$ are given with respect to the reference isotope $^{147}$Pm. For details on fixed and dependent parameters see text. All values are given in units of MHz

Isotope	$\delta \nu ^{147, A^\prime }_{452}$	$\mathcal {A}^l_{452}$	$\mathcal {B}^l_{452}$	$\mathcal {A}^u_{452}$	$\mathcal {B}^u_{452}$	$\delta \nu ^{147, A^\prime }_{468}$	$\mathcal {A}^l_{468}$	$\mathcal {B}^l_{468}$	$\mathcal {A}^u_{468}$	$\mathcal {B}^u_{468}$
$^{147}$Pm	0	620.3(14)	$-$407(18)	500.0(14)	$-$119(15)	0	619.4(17)	$-$407$^*$	438.2(15)	$-$48(13)
$^{146}$Pm	226(12)	429.8(22)	8(19)	347.1(22)	2$^\dagger$	$-$164(16)	429.7(40)	8$^*$	302.6(24)	1$^\dagger$
$^{145}$Pm	344(10)	1255.7(11)	$-$135(17)	1011.7(15)	$-$40$^\dagger$	$-$216(11)	1254.8(15)	$-$135$^*$	886.8(13)	$-$16$^\dagger$
$^{144}$Pm	594(10)	329.0(12)	131(13)	265.2(11)	39$^\dagger$	$-$384(13)	322.0(56)	131$^*$	227.6(46)	15$^\dagger$
$^{143}$Pm	737(11)	1368.9(25)	$-$47(18)	1104.0(28)	$-$14$^\dagger$	$-$495(14)	1363.9(29)	$-$47$^*$	964.8(23)	$-$6$^\dagger$

$*$Fixed parameter; $\mathcal {B}_{468}^l$ set to the value of $\mathcal {B}_{452}^l$

$^\dagger$Dependent parameter; $\mathcal {B}^l/\mathcal {B}^u$ set to the result for $^{147}$Pm

The final values for isotope shifts $\delta \nu$, as well as $\mathcal {A}$- and $\mathcal {B}$-parameters are given in Table 2. The resulting $\mathcal {A}^l/\mathcal {A}^u$ ratios for both transitions remained within the statistical uncertainty ($\mathcal {A}^l_{452}/\mathcal {A}^u_{452} = 1.2401(2)$, $\mathcal {A}^l_{468}/\mathcal {A}^u_{468} = 1.455(10)$). From the two independent parameters $\mathcal {A}^l_{452}$ and $\mathcal {A}^l_{468}$, which are also expected to be identical as both transitions couple to the atomic ground state, we estimate an additional error of $1 \, \text {MHz}$ for all $\mathcal {A}$ hyperfine coupling constants (which is included in Table 2). Similarly, for the $\mathcal {B}$ hyperfine coupling constants, an additional error of $10 \, \text {MHz}$ was added, based upon fit deviations in the $468 \, \text {nm}$ transition with free $\mathcal {B}^l_{468}$-parameters. Note that in these fits the $\mathcal {A}^l_{468}$ changed by much less than $1 \, \text {MHz}$ compared to the fits with $\mathcal {B}^l_{468}$ fixed to $\mathcal {B}^l_{452}$. We did not include the results for $\mathcal {B}^l_{468}$ in Table 2, since we deem $\mathcal {B}^l_{452}$ parameters to be much more accurate. Looking at the values in Table 2, we observe a perfect agreement of $\mathcal {A}^l_{452}$ with $\mathcal {A}^l_{468}$ for the isotopes $^{145,146,147}$Pm. In $^{143,144}$Pm, on the other hand, there is a deviation of few MHz. Considering the rather large uncertainty in $\mathcal {A}^l_{468}(^{144}\mathrm {Pm})$, this deviation is covered within $1.3 \, \sigma$. In $^{143}$Pm, the error margins of $\mathcal {A}^l_{452}$ and $\mathcal {A}^l_{468}$ barely overlap. The significance of these deviations is therefore rather low, but should be noted.

**Table 3:** Nuclear spins $I^\pi$, magnetic dipole moments $\mu _I$ and electric quadrupole moments $Q_s$ for several Pm nuclei. Values with no leading sign indicate that only an absolute value is known. The results for $\mu _I$ and $Q_s$ are calculated according to Eq. 1, with the reference isotope $^{147}$Pm. References for literature values of $\mu _I^\text {lit}$ are given in the last column for the individual isotopes. The values from the reference for $^{144}$Pm and $^{147}$Pm were re-evaluated in this work, for details see text. The values for $^{145}$Pm and $^{149m}$Pm are based on the new reference value of $^{147}$Pm. The Schmidt limits for a $g_{7/2}$ and a $d_{5/2}$ proton are $\mu _I^S(g_{7/2}) = 1.72 \, \mu _N$ and $\mu _I^S(d_{5/2}) = 4.79 \, \mu _N$, respectively. All literature values of $Q_s^\text {lit}$ are taken from [36]

Isotope	*N*	$I^{\pi }$	$\mu _I\, (\mu _\text {N})$	$Q_s\, (e\text {b})$	$\mu _I^\text {lit} \, (\mu _\text {N})$	$Q_s^\text {lit} \, (e\text {b})$	References
			This work	This work	Literature	Literature	
$^{151}$Pm	90	${5}/{2}^{+}$			1.8(2)	2.2(9)	[6]
$^{149m}$Pm	88	${5}/{2}^{+}$			2.0(2)		[37]
$^{149}$Pm	88	${7}/{2}^{+}$			3.3(5)		[38]
$^{148}$Pm	87	${1}^{-}$			+2.1(2)	+0.2(2)	[38]
$^{147m}$Pm	86	${5}/{2}^{+}$			3.53(6)$^*$		[39]
$^{147}$Pm	86	${7}/{2}^{+}$			+2.57(4)$^*$	+0.74(20)	[12]
$^{146}$Pm	85	$3^{-}$	+1.53(3)	$-$0.01(4)			
$^{145}$Pm	84	${5}/{2}^{+}$	+3.72(5)	+0.25(7)	+3.71(5)$^*$	+0.23(8)	[10]
$^{144}$Pm	83	$5^{-}$	+1.95(4)	$-$0.24(7)	1.71(14)$^*$		[40]
$^{143}$Pm	82	${5}/{2}^{+}$	+4.05(6)	+0.08(4)	3.8(5)		[38]

*Re-evaluated results. For details see text

## Results and discussion

### Nuclear moments

**Fig. 6:**
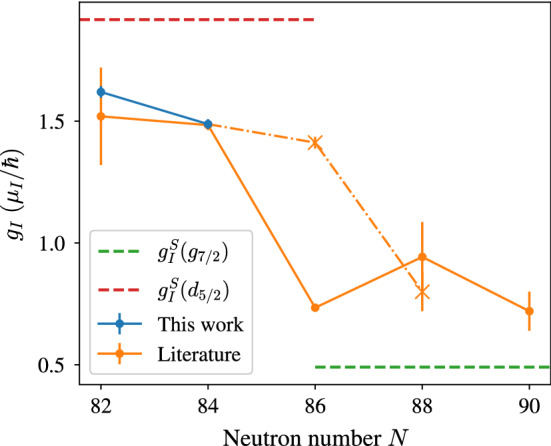
$g_I$ factors for the even-neutron-number Pm isotopes. The dashed lines mark the Schmidt limits of the $d_{5/2}$ and $g_{7/2}$ shell model configurations. The cross markers indicate the isomers $^{147m}$Pm and $^{149m}$Pm at excitation energies of $91 \, \mathrm {keV}$ and $114 \, \mathrm {keV}$, respectively. They are connected to the $^{145}$Pm ground state by the dash-dotted line to guide the eye

Magnetic dipole moments and electric quadrupole moments for Pm isotopes are given in Table 3. Earlier values reported in literature are included. The most precise values are available for $^{147}$Pm, which were measured with different complementary methods, i.e. paramagnetic resonance of Pm IV [41], ABMR of Pm I [6] and optical spectroscopy of Pm II [12], all with similar precision. The result of $\mu _I^\text {lit}(^{147}\text {Pm})=2.58(7) \mu _N$ given in the work of Reader et al. is based on the evaluation of the Gouldsmit–Fermi–Segré formula [42]. The authors used a magnetic splitting factor of $\mathcal {A}=647(13) \, \text {MHz}$ for the $4f^56s^2 \, ^6H_{5/2}$ atomic ground state, which is not a direct experimental result, but was estimated on the basis of experimental data for the $4f^56s^2 \, ^6H_{7/2}$ state from the ABMR measurements in [6]. When we re-evaluate the Gouldsmit–Fermi–Segré formula in Reader’s work, but with our experimental value of $\mathcal {A}=619.9(7) \, \text {MHz}$ (the weighted average of $\mathcal {A}_l^{452}$ and $\mathcal {A}_l^{468}$) for the atomic ground state splitting, the result for the magnetic moment changes to $\mu _I^\text {lit}(^{147}\text {Pm})=2.51(5) \mu _N$. The other values for $\mu _I$ from [41] and [6] were re-evaluated in the scope of [12], based on more recent theoretical results of $\langle r^{-3} \rangle$. Since there is no reason to prefer one of these experimental values over the other, we calculate the weighted average $\mu _I^\text {lit}(^{147}\text {Pm})=2.57(4) \mu _N$. For the electric quadrupole moment, we take the value of $Q_s^\text {lit}(^{147}\text {Pm}) = 0.74(20) \, e\text {b}$ from [36] as reference. It is based on the laser spectroscopy measurements of Alkhazov et al. [43], but takes into account more recent results for the electric field gradient at the location of the nucleus of Pyykkö [44], which no longer rely on Sternheimer corrections. In order to determine nuclear moments $\mu _I$ and $Q_s$ for $^{143-146}$Pm, we use the relations

1 $$\begin{aligned}&\mu _I= \frac{\mathcal {A}}{\mathcal {A}_\text {ref}}\frac{I}{I_\text {ref}}\mu _{I,\text {ref}} \end{aligned}$$

2 $$\begin{aligned}&Q_s= \frac{\mathcal {B}}{\mathcal {B}_\text {ref}}Q_{s\text {,ref}} \end{aligned}$$

with $^{147}$Pm as reference isotope. The results are given in Table 3. With the exception of $\mu _I(^{144}\text {Pm})$, the obtained values agree with the ones previously reported in literature. In the case of $^{144}$Pm, the magnetic dipole moment was determined from the temperature dependence of low temperature nuclear orientation measured via anisotropy of the $\gamma$-radiation of oriented $^{144}$Pm nuclei [40]. If we re-evaluate their experimental result using the same value for $\langle r^{-3} \rangle$ as was used in [12], we obtain $|\mu _I^\text {lit}|(^{144}\text {Pm}) = 1.71(14) \, \mu _N$ (assuming an unchanged uncertainty), which approaches the result from our work, but still lies outside its uncertainty. One might consider a hyperfine anomaly $^i\varDelta ^j$ in this case, which specifies a relative deviation from Eq. 1, i.e. $\mathcal {A}^{i}/\mathcal {A}^{j} = g_I^i/g_I^j (1-\,^i\varDelta ^j)$, with $g_I = -\mu _I/I$ the gyromagnetic ratio for the respective isotopes *i*, *j*. However, the hyperfine anomaly is usually in the order of $<1$% [45], and should thus be covered within the given uncertainty of $\mu _I$ (in order to explain this deviation a hyperfine anomaly of $^{147}\varDelta ^{144}=0.12(7)$ would be required). Also note that the difference of the $\mathcal {A}^l_{452}$ and $\mathcal {A}^l_{468}$ hyperfine coupling constants in $^{144}$Pm, as mentioned in Sect. 3, is covered by the uncertainty of $\mu _I$ and therefore not sufficient to explain this large discrepancy with literature. Since our results for $\mu _I(^{145}\text {Pm})$ are in perfect agreement with laser spectroscopy measurements of Alkhazov et al. [10] (both for $\mu _I$ and $Q_s$), who studied a different transition in singly ionized Pm, we are confident with our results and expect an inconsistency between the literature values of $\mu _I^\text {lit}(^{147}\text {Pm})$ and $\mu _I^\text {lit}(^{144}\text {Pm})$, with the former being used as reference for the values in our work. Looking at the even-neutron-number isotopes, one clearly observes the expected trend of increasing deformation from a rather spherical nucleus at the magic neutron number $N=82$ towards more neutron rich isotopes. The trend in $g_I$ factors is plotted in Fig. 6. Due to their small deformation, $^{143}$Pm and $^{145}$Pm can be represented by the $d_{5/2}$ shell model state. They lie close to the Schmidt limit of $g_I^S(d_{5/2}) = 1.92 \, \mu _N/\hbar$. Adding more neutrons changes this situation. After a small decrease of $g_I$ from $^{143}$Pm to $^{145}$Pm, a sudden drop is observed towards $^{147}$Pm. As can be seen in Fig. 6, $^{147,149,151}$Pm lie closer to the Schmidt limit of $g_I^S(g_{7/2}) = \, 0.49 \mu _N/\hbar$. This can be explained by a positive (prolate) quadrupole deformation, leading to the population of the 7/2[404] Nilsson orbital in $^{147,149}$Pm and the 5/2[413] orbital in $^{151}$Pm [46], both belonging to the $g_{7/2}$ shell model state. The same transition can be observed in the isomeric states $^{147m}$Pm at $91 \, \mathrm {keV}$ and $^{149m}$Pm at $114 \, \mathrm {keV}$ excitation energy, but shifted by two *N*, indicating that the $d_{5/2}$ states are located at increasingly high excitation energy. In the quadrupole moments, the increasing deformation is even more clearly visible. The unpaired proton induces a prolate ($Q_s> 0$) deformation as neutrons are added, with the trend becoming very steep towards $^{151}$Pm [6]. For the odd-neutron-number isotopes, on the other hand, the unpaired neutron induces an oblate ($Q_s< 0$) deformation, which to some degree compensates the one from the unpaired proton, but is much less pronounced. Consequently, $^{144}$Pm exhibits a negative quadrupole moment of $Q_s= -0.24(7) \, e\text {b}$, increasing to $Q_s= -0.01(4) \, e\text {b}$ for $^{146}$Pm, where the deformation induced by the single proton and neutron states compensate, and continues to larger positive quadrupole moments for $^{147,151}$Pm, where the influence of the valence proton becomes dominant.

### Isotope shifts

The isotope shift defines the frequency difference $\delta \nu$ in an atomic transition *i* for two isotopes with mass numbers *A* and $A'$,

3 $$\begin{aligned} \delta \nu _i ^{A,A'} = \nu _i^A - \nu _i ^{A'}. \end{aligned}$$

Our measured results for $^{143-147}$Pm are included in Table 2, extracted from the center of gravity of the individual hyperfine structures relative to the center frequency of $^{147}$Pm. The data shows a different sign for isotope shifts in the $452 \, \text {nm}$ and the $468 \, \text {nm}$ transition, i.e. $\delta \nu _{452}^{A,A'} > 0$ and $\delta \nu _{468}^{A,A'} < 0$ for $A>A'$. The sign of the isotope shift gives hints on the configuration of the upper levels in the respective transition. For the $468 \, \text {nm}$ transition, we expect a $4f^56s^2 \rightarrow 4f^56s6p$, since the involved *s* electron requires a higher transition frequency for lighter (i.e. smaller) nuclei. From the positive isotope shifts in the $452 \, \text {nm}$ transition we conclude that no *s* electron is involved and expect a $4f^56s^2 \rightarrow 4f^45d6s$ transition. However these assignments are based on the expected change in electron density at the nucleus and should be used with care.

In order to analyze the data with regard to changes in mean square charge radii, one can express the isotope shift as

4 $$\begin{aligned} \delta \nu _i ^{A,A'} = \delta \nu ^{A,A'} _{i, M} + \delta \nu ^{A,A'} _{i, F} = K_i \frac{1}{\mu ^{A,A'}} + F_i \delta \langle r^2 \rangle ^{A,A'} \end{aligned}$$

where $\delta \nu ^{A,A'} _{i, M}$ denotes the mass shift and $\delta \nu ^{A,A'} _{i, F}$ the field shift, accounting for the change of mass and volume of the nucleus, respectively. They depend on the mass shift constant $K_i$, the reduced mass $\mu ^{A,A'}=m_Am_{A'}/(m_A-m_{A'})$, the field shift constant $F_i$ and the change in the mean square charge radius $\delta \langle r^2 \rangle ^{A,A'}$ between two isotopes with mass numbers *A* and $A'$. Both, $K_i$ and $F_i$ depend on the atomic transition and have to be carefully analyzed in order to quantitatively extract $\delta \langle r^2 \rangle ^{A,A'}$. In our case this analysis is hampered by an uncertain assignment of the excited atomic levels and the lack of theory input. Still, from the data of well-studied neighboring elements and the fact that we have measured isotope shifts in two atomic transitions, an evaluation may be attempted.

**Fig. 7:**
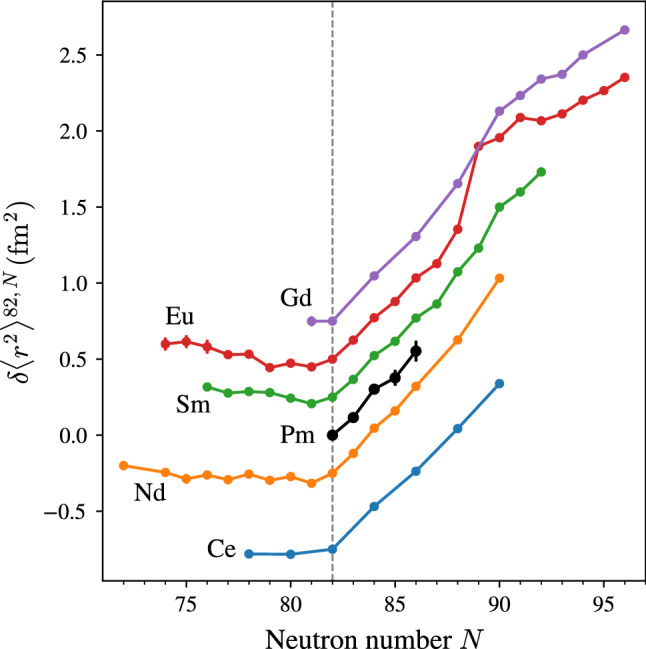
Changes in mean square charge radii in the promethium region. Data for the neighboring elements is taken from [47]. Arbitrary offsets of multiples of $0.2 \, \text {fm}^2$ are added to the different isotopic chains for visual separation. For details see text

The mass shift can be further separated to the so-called normal mass shift (NMS) and the specific mass shift (SMS), accounting for the change in the center of motion and electron-electron correlations, respectively. While the former can be exactly calculated via $\text {NMS}_i = \nu _i/1836.1$ [42], estimations about the SMS require theory input. However, the SMS is often in the order of the NMS, and we thus assume $\text {SMS}_i = 0 \pm \text {NMS}_i$, as often done in cases where SMS$_i$ is not known [1]. With Eq. 4 we can then calculate the field shift $\delta \nu ^{A,A'} _{i, F}$, which usually dominates the isotope shift in heavy atoms. In order to extract values for $F_i$ and $\delta \langle r^2 \rangle ^{A,A'}$, we rely on data of neighboring elements, as compiled in [47]. In the reference, data from *K* X-ray shifts, elastic electron scattering, muonic atoms and optical isotope shifts is evaluated for the extraction of root mean square charge radii. However, the listed changes in rms charge radii $\delta \langle r^2 \rangle ^{A,A'}$ only take optical isotope shifts into account. The following analysis is based on the latter. Starting from the neutron shell closures at $N=28,50,82$ and 126, the increase in nuclear charge radii is approximately linear towards neutron-rich isotopes and neighboring elements exhibit similar slopes, with few exceptions. Assuming this trend holds valid for Pm, we can use this regularity to extract the field shift constant $F_i$. The mean square charge radii of the Pm neighbors Ce, Nd, Sm, Eu and Gd over the even neutron number isotopes, i.e. $N=82,84,86$ is given in Table 4. As a weighted average we obtain $\overline{\delta \langle r^2 \rangle ^{N,N+2}} = 0.276(13) \, \text {fm}^2$. The field shift constant $F_i$ of the two investigated transitions is varied until the change in mean square charge radii, related to $F_i$ with Eq. 4, matches this value. We obtain

$$\begin{aligned} F_{452}&= -1210(60) \, \text {MHz/fm}^2\\ F_{468}&= +1015(55) \, \text {MHz/fm}^2 \end{aligned}$$

in the $452 \, \text {nm}$ and $468 \, \text {nm}$ transition, respectively, where the uncertainty is derived from the standard deviation of $\delta \langle r^2 \rangle ^{N,N+2}$ in Table 4. The results, together with the data of the neighboring elements from [47], are displayed in Fig. 7.

**Table 4:** Change in mean square charge radius per two neutrons for different light lanthanide elements, evaluated for neutron numbers $N=82,84,86$. Nuclear charge radii data is taken from [47]

*Z*	Element	$\delta \langle r^2 \rangle ^{N,N+2} \, (\text {fm}^2)$
64	Gd	0.282(8)
63	Eu	0.272(12)
62	Sm	0.274(22)
60	Nd	0.296(20)
59	Ce	0.256(14)

The trend in charge radii exhibits several interesting features. Most noticeable is the kink in the curve of all elements at the magic neutron number $N=82$, followed by a similar slope in $\delta \langle r^2 \rangle ^{A,A'}$ for all displayed elements with increasing neutron number. Below $N=82$, a *Z*-dependence in the slope can be observed. While Ce and Nd charge radii are rather constant, Sm and Gd show increasing charge radii towards neutron deficient isotopes. Pm lies exactly in between these two trends, which highly motivates further measurements in this region. Note that an even more distinct *Z*-dependence is observed around the $N=28$ shell closure, which is discussed e.g. in [48]. Lastly, one should note the sudden increase in the charge radius of Eu from $N=88$ to $N=89$, which has been related to the influence of the almost-doubly magic $^{146}$Gd [5].

In order to verify the results of the neighboring elements analysis and to obtain a reasonable error estimate on $F_i$, and accordingly on $\delta \langle r^2 \rangle ^{A,A'}$, a complementary King-plot analysis was performed. A King–Plot can be used to determine $F_{468}/F_{452}$ with just the isotope shifts as underlying data, i.e. independent of the assumptions made above. The relation between the modified isotope shifts $\mu \delta \nu ^{A,A'}$ in different atomic transitions *i*, *j* is expected to be linear up to high precision, and can be expressed as

5 $$\begin{aligned} \mu \delta \nu _i ^{A,A'} = \frac{F_i}{F_j}\mu \delta \nu _j^{A,A'} + \left( K_i - \frac{F_i}{F_j}K_j \right) . \end{aligned}$$

**Fig. 8:**
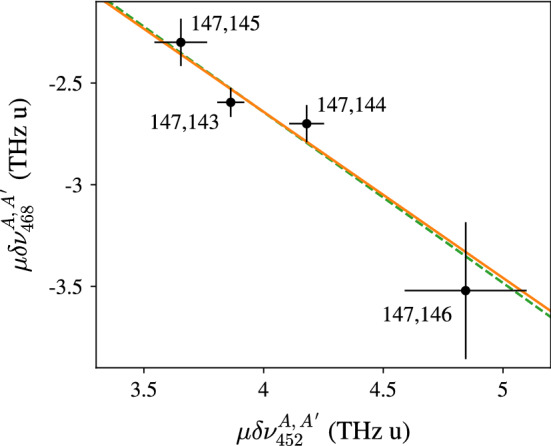
King–Plot analysis of the investigated optical ground-state transitions at 452 nm and 468 nm. The modified isotope shifts are plotted with respect to the reference isotope $^{147}$Pm. The solid orange line displays the best fit to the data and the green dashed line has a fixed slope of $F_{468}/F_{452}=0.84$, as obtained from the analysis of neighboring elements

The ratio of the respective field shift constants is given by the line slope. The King–Plot of the modified $452 \, \text {nm}$ and $468 \, \text {nm}$ isotope shifts is presented in Fig. 8. The best fit to the data yields a slope of $F_{468}/F_{452} = -0.82(24)$, in excellent agreement with the value of $F_{468}/F_{452} = -0.84(6)$ from the neighboring element analysis. Note that using this ratio from the King–Plot, the $F_i$ values would be shifted apart from each other by $\approx 30 \, \text {MHz}$, well within the stated uncertainty from the neighboring element analysis. For the mass shift constants $K_i$, on the other hand, the King–Plot analysis is less conclusive, since the uncertainty of the offset is in the order of $150 \, \%$ of the value itself and consistent with zero. From the best fit we can extract

$$\begin{aligned} K_{468}+0.82(24) \cdot K_{452} = 620(940) \, \text {GHz/u} \end{aligned}$$

which agrees with our assumption of

6 $$\begin{aligned} \text {NMS}_{468}+0.84(6) \cdot \text {NMS}_{452} = 640(14) \, \text {GHz/u} \end{aligned}$$

but prevents any reasonable conclusions about the individual components. Note that the uncertainty in (6) would increase to $460 \, \text {GHz/u}$ when the assumption $\text {SMS}_i = 0\, \pm \, \text {NMS}_i$ would have been considered.

**Table 5:** Changes in mean square charge radii with respect to $^{147}$Pm. The values derived from isotope shifts in the $452 \, \text {nm}$ and the $468 \, \text {nm}$ transitions are averaged, with uncertainties in brackets. The last column contains the staggering parameter $\gamma _A$ for the odd-neutron-number isotopes

*A*	$\delta \langle r^2 \rangle ^{147,A}_\text {avg} \, (\text {fm}^2)$	$\gamma _A$
146	$-$0.17(2)	0.60(17)
145	$-$0.24(3)	
144	$-$0.42(5)	0.77(15)
143	$-$0.53(6)	

Finally, we extract values for the changes in nuclear mean square charge radii, as given in Table 5. From the values of $\delta \langle r^2 \rangle ^{147,A}_{452}$ and $\delta \langle r^2 \rangle ^{147,A}_{468}$, individually derived from the isotope shifts in the transitions under investigation, we calculated an average $\delta \langle r^2 \rangle ^{147,A}_\text {avg}$. The results indicate an odd-even staggering in mean square charge radii, which is defined via

7 $$\begin{aligned} \gamma _A = \frac{2\delta \langle r^2 \rangle ^{A-1,A}}{\delta \langle r^2 \rangle ^{A-1,A+1}} \end{aligned}$$

for odd-neutron-number isotopes, with the staggering parameter $\gamma _A$ [49]. For $\gamma _A < 1$, this is referred to as normal odd-even staggering. It is qualitatively related to the quadrupole deformation, as given in Table 3. In $^{144}$Pm, $\gamma _A$ is closer to 1 (little staggering), since the deformation in $^{144,145}$Pm is similar, despite the different sign in $Q_s$. $^{146}$Pm, however, is almost spherical due to the compensating deformation induced by the single proton and the single neutron orbitals, whereas in particular the neighboring $^{147}$Pm has a rather high quadrupole moment, resulting in a staggering parameter significantly different from 1.

## Summary

We measured hyperfine spectra of the five long-lived promethium isotopes $^{143-147}$Pm in two different atomic ground state transitions, allowing the precise extraction of hyperfine coupling constants and isotope shifts. From this data, refined values for the magnetic moments of $^{143,144,145}$Pm were extracted. The magnetic moment of $^{146}$Pm and quadrupole moments of $^{143,144,146}$Pm were determined for the first time. Since the excited state configuration in the transitions under investigation is unknown, a precise analysis of the isotope shifts was hampered. However, a comparison with neighboring elements allowed good estimate on the transitions’ field shift constants and changes in mean square charge radii. The results indicate that the specific mass shift in both transitions is small compared to the field shift. A King–Plot analysis confirmed the consistency of our results and allowed an estimate of systematic uncertainties.

For a better understanding of the evolution of deformation, visible in nuclear moments and changes in mean square charge radii, it is of high relevance to continue laser spectroscopy studies of Pm, both towards $^{151}$Pm, but also of light Pm nuclei below $N=82$. With this work we established a basis for future experiments aiming for more exotic nuclei at radioactive ion beam facilities, possibly by using the PI-LIST ion source, which has recently been adapted for on-line application.

## Data Availability

This manuscript has no associated data or the data will not be deposited. [Author’s comment: comment: The spectroscopic data of all laser scans which were performed within the scope of this work are available upon request to the corresponding author. All other relevant data is included in the presented manuscript.]

## Footnotes

1 In this context, on-line means the experiment is coupled to the isotope production site, enabling experiments on short-lived nuclei.
